# Is it time to rethink how we measure the quality of life in young-onset dementia?

**DOI:** 10.1177/13872877251385197

**Published:** 2025-10-09

**Authors:** Aderonke Agboji, Darryn DiFrancesco, Komla Avoumatsodo

**Affiliations:** 1 School of Nursing, University of Northern British Columbia, Quesnel, British Columbia, Canada; 2 School of Nursing, University of Northern British Columbia, Fort St John, British Columbia, Canada; 3 School of Economics, University of Northern British Columbia, Prince George, British Columbia, Canada

**Keywords:** Alzheimer's disease, dementia, longitudinal studies, quality of life, young onset dementia

## Abstract

Young-onset dementia (YOD) presents distinct psychological, relational, and practical challenges. In their longitudinal study, Aspo et al. examine self-reported quality of life in people with YOD over two years, revealing group-level stability alongside notable individual variation. These findings challenge deficit-focused views of dementia and highlight the need for early, sustained support that preserves agency and identity. This commentary situates the study within clinical practice, calling for greater attention to the lived experiences behind quantitative measures and emphasizing the importance of relational and narrative-informed approaches in YOD care.

Reading the study by Aspo et al.^1^ reminded us of the importance of listening not just to what people say, but also how they say it, and sometimes, what they are not able to say. The study follows 33 individuals over a two-year period following a diagnosis of young-onset dementia (YOD), tracking how they rate their quality of life (QoL) and health-related quality of life (HRQoL) using established instruments including QoL-AD and RAND-36. The findings indicate a marked stability in self-reported QoL at the group level. At the same time, the authors highlight considerable variation in individual experiences beneath these group averages. In our view, this variability underscores the capacity of individuals with YOD to adapt in nuanced and personally meaningful ways. While we do not suggest that the field still views dementia as a uniform trajectory of decline, the continued reliance on standardized measures can inadvertently perpetuate older framings of dementia as a straightforward decline, thereby obscuring the lived variability that Aspo et al.^1^ so clearly demonstrate.

We particularly appreciated the study's commitment to self-reporting, a choice that reflects both ethical sensitivity and clinical realism. As a health care provider who works directly with patients navigating early dementia diagnoses (AA), and the family members of someone experiencing dementia (DD and KA), we cannot overstate how crucial it is to ask individuals how they themselves are experiencing their lives. In research and care alike, proxy reports often substitute for the voices of those living with dementia, especially as cognitive symptoms progress. But in early stages, people with YOD can reflect insightfully on their needs, emotions, and values.^2^ Elevating their voice is not simply a gesture of inclusion, it is a clinical imperative. Self-reporting enables us to capture invisible domains of distress, such as changes in social roles, emotional fatigue, or loss of identity, which standardized clinical observations may miss.^2^

That said, we found ourselves questioning the tools we use to elicit these self-reports. While QoL-AD and RAND-36 are validated instruments, they are structured to yield data, not depth. In our experience, a stable score does not always mean stable well-being. A participant may check a box that says “moderate” or “somewhat satisfied,” and yet moments later describe feelings of confusion, grief, or isolation. These are not inconsistencies; they are truths that do not always fit the confines of structured scales. In this way, we worry that the apparent stability in scores might mask tremendous cognitive and emotional labor.

People with YOD often work hard to maintain roles, routines, and a sense of self long before they articulate distress. By the time a clinical score changes, the underlying loss may already be well underway. This raises a broader concern about how we, as health professionals and researchers, conceptualize QoL. It is tempting to equate stability in scores with resilience, but we would argue that resilience is often embedded in the struggle, not just the outcome. A person may report “doing fine,” while privately mourning a role lost at work, or feeling excluded in family decision-making. They may also report “doing fine” after recalibrating their expectations around their condition, rather than against their previous baseline of well-being. Tools such as QoL-AD and RAND-36 offer valuable baselines, but they rarely capture the full texture of daily life with dementia.^2^ This is especially true when we consider the variation in individual experiences that the group averages conceal.

While the value of qualitative QoL research in dementia is well established, standardized instruments continue to dominate research reporting and clinical practice,^3^ often relegating qualitative findings to a supplementary role. For this reason, we believe that QoL assessments, particularly in YOD, should be complemented with open-ended, narrative-based approaches.^4^ A brief conversation about meaning, identity, or fear can often reveal more than a numerical score. Narrative offers space for contradiction allowing someone to say, “I’m doing okay” and “I feel lost” in the same breath. This is not confusion, but coherence in the face of instability. Studies such as Aspo et al.'s^1^ provide a vital foundation.

We recognize that Aspo et al.^1^ included qualitative interviews and explicitly acknowledged their value. Our concern, however, is that their conclusions placed greater emphasis on improving standardized instruments, which risks relegating qualitative data to a secondary role. We argue instead that narrative accounts should be treated as central evidence rather than supplementary materials. Building on this, we recommend that a multi-dimensional approach that integrates quantitative measures with qualitative accounts from both individuals and caregivers would provide a stronger, triangulated framework for capturing the complexity and dynamic nature of QoL.^5^

There are, of course, limitations to this study. The sample was limited to Swedish participants and may not reflect more diverse cultural perspectives on aging, memory, and identity. Given Sweden's robust social safety net and healthcare system,^6^ these findings may not translate to contexts with fewer structural supports. Research that incorporates race, socioeconomic status, and varying policy environments could reveal how broader systems shape individual's quality of life. Additionally, while the longitudinal design is a strength, future work might incorporate qualitative data alongside quantitative measures to better understand the complex interplay between self-perception and emotional well-being over time. Nonetheless, the value of this study lies in its clear affirmation of the individual voice in dementia care and its demonstration that people with YOD remain active agents in shaping their own lives.

In conclusion, we commend Aspo et al.^1^ for a sensitive, well-constructed study that contributes meaningfully to our understanding of living well with YOD. As practitioners, we must treat qualitative accounts as central evidence, not relegated to a subsidiary role. Standardized instruments can provide baselines, but they should always be paired with narrative approaches in clinical assessment. Without this integration, we risk missing the cognitive, emotional, and relational work that sustains patients’ lives behind seemingly stable scores. Embedding stories as essential data is therefore not optional but necessary for delivering optimal care for individuals with YOD.

## References

[1] Aspo M Visser L Seiger Cronfalk B , et al. Quality of life of persons with young onset dementia: Repeated self-reported assessment over two years from diagnosis. J Alzheimers Dis 2025, Epub ahead of print. 29 August 2025; DOI: 10.1177/13872877251371305. PMC12495120 40879411

[2] Wu YT Nelis SM Quinn C , et al. Factors associated with self- and informant ratings of quality of life, well-being and life satisfaction in people with mild-to-moderate dementia: results from the improving the experience of dementia and enhancing active life programme. Age Ageing 2020; 49: 446–452. 32037460 10.1093/ageing/afz177

[3] Bowling A Rowe G Adams S , et al. Quality of life in dementia: a systematically conducted narrative review of dementia-specific measurement scales. Aging Mental Health 2014; 19: 13–31. 24881888 10.1080/13607863.2014.915923

[4] Spreadbury JH Kipps C . Measuring younger onset dementia: what the qualitative literature reveals about the ‘lived experience’ for patients and caregivers. Dementia (London) 2019; 18: 579–598. 28114802 10.1177/1471301216684401

[5] Moyle W Murfield JE Griffiths SG , et al. Assessing quality of life of older people with dementia: a comparison of quantitative self-report and proxy accounts. J Adv Nurs 2012; 68: 2237–2246. 22211637 10.1111/j.1365-2648.2011.05912.x

[6] Ludvigsson JF Bergman D Lundgren CI , et al. The healthcare system in Sweden. Eur J Epidemiol 2025; 20: 563–579. 10.1007/s10654-025-01226-9 PMC12170770 40383868

